# Infant AFAR: Automated facial action recognition in infants

**DOI:** 10.3758/s13428-022-01863-y

**Published:** 2022-05-10

**Authors:** Itir Onal Ertugrul, Yeojin Amy Ahn, Maneesh Bilalpur, Daniel S. Messinger, Matthew L. Speltz, Jeffrey F. Cohn

**Affiliations:** 1 Utrecht University, Utrecht, The Netherlands; 2 University of Miami, Miami, FL USA; 3 University of Pittsburgh, Pittsburgh, PA USA; 4 University of Washington, Seattle, WA USA

**Keywords:** Automatic facial action unit detection, Facial action coding system, Infant behavior, Cross domain generalizability, Deep learning

## Abstract

Automated detection of facial action units in infants is challenging. Infant faces have different proportions, less texture, fewer wrinkles and furrows, and unique facial actions relative to adults. For these and related reasons, action unit (AU) detectors that are trained on adult faces may generalize poorly to infant faces. To train and test AU detectors for infant faces, we trained convolutional neural networks (CNN) in adult video databases and fine-tuned these networks in two large, manually annotated, infant video databases that differ in context, head pose, illumination, video resolution, and infant age. AUs were those central to expression of positive and negative emotion. AU detectors trained in infants greatly outperformed ones trained previously in adults. Training AU detectors across infant databases afforded greater robustness to between-database differences than did training database specific AU detectors and outperformed previous state-of-the-art in infant AU detection. The resulting AU detection system, which we refer to as Infant AFAR (Automated Facial Action Recognition), is available to the research community for further testing and applications in infant emotion, social interaction, and related topics.

## Introduction

Prior to the development of speech, communication depends on nonverbal behavior. Facial actions are a primary means for infants to communicate their emotions and intentions and regulate social interaction. The most comprehensive method to annotate facial actions is the anatomically based Facial Action Coding System (FACS) (Ekman, Friesen, & Hager, 2002; Cohn & Ekman, 2005). FACS action units (AUs) are actions of individual or a group of facial muscles. For example, AU12 (lip corner puller) is caused by contraction of the zygomatic major muscle, that pulls the lip corners obliquely (Cohn & Sayette, 2010). Alone or in combinations, AUs can describe most facial expressions with respect to component actions. Inferring emotion from facial movements and universality of facial expressions may be controversial (Barrett, Adolphs, Marsella, Martinez, & Pollak, 2019; Cowen et al., 2021), but not the descriptive scope of FACS. Unlike systems that use emotion labels to describe expression, FACS explicitly distinguishes between facial actions and inferences about what they mean. Inferences about the emotional meaning of facial actions are extrinsic to FACS (Cohn, Ambadar, & Ekman, 2007).

Baby FACS (Oster, 2006), which is an extension of FACS (Ekman et al., 2002) for infants, is an anatomically-based method to manually annotate facial action units in infant faces. Baby FACS coding, like FACS coding, is labor-intensive, requires expert training, and is ill suited for real-time applications. An automated, objective, reliable system that can work in real-time would enable greatly expanded use of facial action coding in a wide range of applications.

Automated AU detection in infants has numerous current and potential research and clinical uses. Recent applications include investigating how infants cope with changes in their mother’s affect and contingent responsiveness (Ahn et al., 2020a; Ahn, Onal Ertugrul, Chow, Cohn, & Messinger, 2021), infant response to frustration and to stimuli intended to elicit positive emotion (Hammal et al., 2018), and infant responses to different foods (Maroulis, Spink, Theuws, Oster, & Buitelaar, 2017). A validated automated system available to the research community could expand research on these topics and contribute to a variety of additional research questions. These include identifying infants at risk for insecure attachment (Cohn, Campbell, & Ross, 1991; Mesman, van IJzendoorn, & Bakermans-Kranenburg, 2009; Beebe & Steele, 2013) and infants with facial nerve abnormalities (Hammal, Chu, Cohn, Heike, & Speltz, 2017); infant food and taste preferences (Forestell & Mennella, 2017; Rosenstein & Oster, 1988), experience of pain (Kohut, Riddell, Flora, & Oster, 2012; Mattson, Cohn, Mahoor, Gangi, & Messinger, 2013) and response to maternal depression and distress (Campbell, Cohn, & Meyers, 1995). Mother-infant clinical interventions (Beebe, 2020) could be scaled to larger numbers of mothers and infants and to real-time use. Given its many potential uses, automated AU detection in infant faces is under-studied.

Unlike in infants, automated detection of AUs in adult faces has been widely studied. Early studies in infants used what are referred to in machine learning as “shallow approaches” in which facial features are extracted and then used to train classifiers. Facial features include appearance features that describe the texture or color of facial regions (Jiang, Valstar, Martinez, & Pantic, 2014; Chen, Liu, Tu, & Aragones, 2013; Baltrusaitis, Zadeh, Lim, & Morency, 2018), geometric features that capture the statistics derived from the location of facial landmarks (e.g., lip corners) (Mahoor, Cadavid, Messinger, & Cohn, 2009) and motion features that capture the deformations in the skin related to facial muscle contraction (Valstar, Pantic, & Patras, 2004). Such features are often referred to as *hand-crafted* in that they are defined a priori. Hand-crafted features are generally combined to train and test AU classifiers such as Support Vector Machines (SVM) (Burges, Burges (1998); Hsu, Chang, & Lin, 2003), and Artificial Neural Networks (ANN) (Hinton, 1992).

By contrast, the most powerful contemporary approach is “deep learning” (LeCun, Bengio, & Hinton, 2015), in which the informative features are learned automatically from the video during training. Several deep methods (Zhao, Chu, & Zhang, 2016; Chu, De la Torre, & Cohn, 2017; Onal Ertugrul, Yang, Jeni, & Cohn, 2019c; Yang et al., 2019) have been proposed and shown to outperform shallow approaches for AU detection.

Most of the available open source or commercial AU detectors are trained with the faces of young adults. OpenFace (Baltrusaitis et al., 2018) and AFAR (Onal Ertugrul, Jeni, Ding, & Cohn, 2019b) are open source toolboxes that both provide a user-friendly GUI and are easy to use by non-programmers. However, AU detectors of both of these tools are trained with databases containing only adult faces. How they generalize to detect AUs in the infant faces is unknown. AFFDEX by Affectiva, FaceReader by Noldus, and CERT/FACET by iMotions are commercial AU detectors and they are either not accessible to all researchers or costly. Moreover, the databases that are used to train these systems as well as their cross-domain generalizability are unknown.

AU detectors trained with adult faces are generally shown to perform well within the same domain (e.g. same or similar experimental conditions such as context, video resolution, illumination, head pose). Yet, they show diminished generalizability to new domains even if the age distributions in both domains are similar (Onal Ertugrul et al., 2019a). Infant faces differ from adult faces in terms of proportion (e.g. larger eyes and smaller jaw-to-face ratio), skin smoothness, amount of texture and wrinkles and presence of brow knitting action (Oster, 2006; Eibl-Eibesfeldt, 1970). For these reasons, AU detectors trained with adult faces may not be well suited to detect actions in infant faces. Models specifically trained to detect AUs in infant faces are needed.

Earlier studies on AU detection in infants used a semi-automatic computer vision approach (Active Appearance Model (Cootes, Edwards, & Taylor, 2001; Matthews & Baker, 2004) to track faces and extract facial features. They required manual initialization and person-specific training (Messinger, Mattson, Mahoor, & Cohn, 2012; Zaker, Mahoor, Messinger, & Cohn, 2014; Mattson et al., 2013). In part for this reason, training and testing were limited to small numbers of infants. Twelve infants was the largest number used; in one case, as few as two infants and two AUs were used (Messinger, Mahoor, Chow, & Cohn, 2009).

More recently, fully automated approaches have been proposed. Baby FaceReader (Maroulis et al., 2017) is a commercial AU detector for use in infants developed by Noldus. Baby FaceReader expanded the number of AUs relative to previous approaches but was validated on a scant 74 video frames. Hammal et al. (2017) proposed a deep approach that uses convolutional neural networks (CNNs) to automatically detect nine AUs in video of infant faces. Because their experiments were limited to a single database, cross-domain generalizability of their models could not be evaluated, there was no comparison of their AU detectors with ones trained in adults, and their infant AU detectors are not publicly available for other researchers to use.

**Fig. 1:**
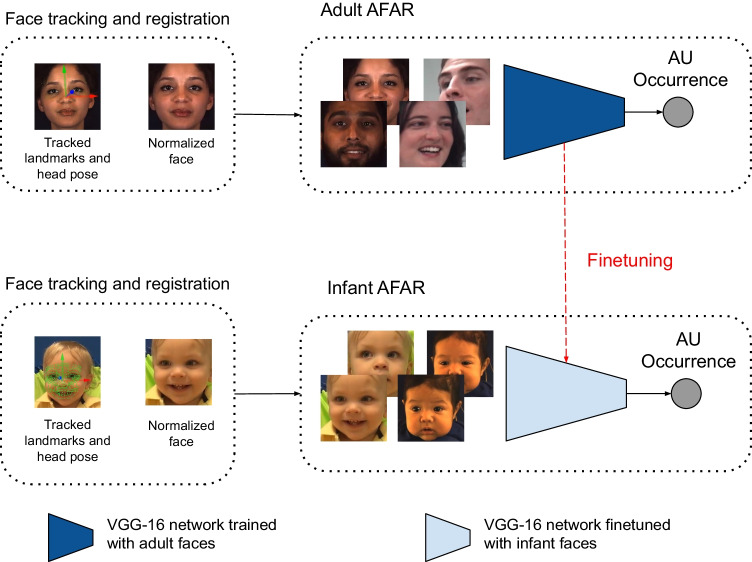
Steps to obtain Infant AFAR. (a) First, a VGG-16 network that is pre-trained on ImageNet database is trained with adult faces to obtain Adult AFAR. (b) Weights of the Adult AFAR network are used to initialize Infant AFAR. Then initialized network is further fine-tuned using infant faces in MIAMI and CLOCK databases to obtain Infant AFAR that can detect AUs in infant faces automatically

Using a deep learning approach to fully automated AU detection in infants and larger databases than used previously, we pursued three related questions:

1. Do AU detectors trained in adult faces generalize to infant faces? We compared state-of-the-art AU detectors trained in adults (Onal Ertugrul et al., 2019b) with ones trained in infants. We refer to the ones trained in adults as Adult AFAR. Based on prior research on generalizability of AU detectors between different domains, we hypothesized that Adult AFAR would perform less well than AU detectors trained specifically in infants.
2. Do AU detectors trained in one infant database generalize to another infant database? Based on prior research, we anticipated that generalizability between infant databases would be attenuated.
3. Does “pre-training” AU detectors in adults and then training on infants afford advantages relative to training infant AU detectors from scratch? In training from scratch, weights of the network are randomly initialized. Earlier work on several computer vision tasks has found that using the weights of a pre-trained neural network and then fine-tuning it (that is, re-training the initialized neural network) generally outperforms training from scratch. Especially when data sets are small, starting with learned weights rather than random ones is helpful. To investigate this, we compared the performances of infant AU detectors trained from scratch with an AU detector that was pre-trained on adult faces and fine-tuned on infant faces.

The findings lead us to propose Infant AFAR, a fully automated tool to detect AUs in video of infants (see Fig. 1). Rather than initializing the weights of Infant AFAR randomly, we use Adult AFAR as the initial neural network and use its weights to initialize our new model, referred to as Infant AFAR. Initial weights capture information about detecting AUs in adults. Then we fine-tune (train the initialized neural network), Infant AFAR, with the faces of infants in two large, well-annotated infant databases for four AUs namely, AU4, AU6, AU12, and AU20. The databases are FF-NSF-MIAMI (referred to as MIAMI for brevity) (Chen, Chow, Hammal, Messinger, & Cohn, 2020; Hammal, Cohn, & Messinger, 2015) and CLOCK (Hammal et al., 2017) which differ in terms of infant age, context, illumination, and video resolution. We also train models for five additional AUs that are manually annotated only in CLOCK; namely, AU1, AU2, AU3, AU9 and AU28, and perform comparisons with the available AU detectors. We make Infant AFAR publicly available to the research community as a part of AFAR toolbox which has a user-friendly GUI for use by non-programmers.

## AU detection in infants

### Databases

We performed experiments using two well-annotated, large infant spontaneous behaviour databases that differ in infant age, context, and video resolution.

**MIAMI** is a database of spontaneous behavior in 43 four-month old infants (Chen et al., 2020; Hammal et al., 2015). Infants were recorded while they interacted with their mothers in a Face-to-Face/Still-Face (FF/SF) protocol (Adamson & Frick, 2003) that elicits both positive and negative affect. FF/SF protocol assesses infant responses to parent unresponsiveness, which is an age-appropriate stressor. The FF/SF has three episodes: (i) parent and infant engage in face-to-face interaction (FF), (ii) the parent stops interacting with the infant and gazes at them with a neutral expression (SF), and (iii) the parent-infant interaction resumes (RE). Video resolution is $1288 \times 964$. In total there are 116K manually annotated frames in 129 videos (of 43 infants for each FF, SF, and RE episodes). AUs were manually annotated from the video by certified FACS (Ekman et al., 2002) coders with advanced training in Baby FACS (Oster, 2006) for four action units: AU4 (brow lowerer), AU6 (cheek raiser), AU12 (lip corner puller), and AU20 (lip stretcher). The combination of AU6 and AU12 is associated with positive affect; AU4 and AU20 with negative affect (Messinger et al., 2012; Camras, 1992; Matias & Cohn, 1993). Inter-observer reliability, quantified using coefficient kappa, averaged 0.85.

The second database was generated by a multisite, longitudinal project known as **CLOCK** (**C**raniofacial microsomia: **L**ongitudinal **O**utcomes in **C**hildren pre-**K**indergarten), which examined the neurodevelopmental and phenotypic outcomes of infants with craniofacial microsomia (CFM) and demographically-matched controls (Luquetti et al., 2019; Speltz et al., 2018). As CFM is characterized by mostly mild, but sometimes severe facial asymmetries (Heike et al., 2016; Hammal et al., 2017), a subset of CLOCK participants (44 cases and 36 controls) was observed and video recorded at age 13 months to compare facial expressiveness across groups (see Hammal et al., 2018). Specifically, two age-appropriate emotion induction tasks were used to elicit spontaneous positive and negative facial expressions (Goldsmith & Rothbart, 1999). In the positive emotion task, an experimenter blew soap bubbles towards the infant. In the negative emotion task, an experimenter presented a toy car to the infant, allowed the child to touch it, then retrieved the car and covered it with a transparent plastic bin. Both tasks were repeated three times unless the infant became too upset to continue or the mother became uncomfortable with the procedure. Each video was approximately 2 min in duration (745K frames and 634K tracked frames in all). Video resolution was 1920 x 1080. AUs were manually annotated from the video by certified FACS coders with advanced training in Baby FACS for nine action units: AU1 (inner brow raiser), AU2 (outer brow raiser), AU3 (inner brows drawn together), AU4 (brow lowered), AU6 (cheek raiser), AU9 (nose wrinkle), AU12 (lip corner puller), AU20 (lip stretcher), and AU28 (lip suck). To assess inter-coder agreement, two or more of the coders independently coded on a frame-by-frame basis 15 seconds of randomly selected segments from 68 infants. Inter-coder agreement, quantified using free-margin kappa (Brennan & Prediger, 1981), was 0.82.

### Automatic face tracking and registration

For automatic face tracking and registration we use the ZFace module (Jeni, Cohn, & Kanade, 2017) of AFAR toolbox (Onal Ertugrul et al., 2019b). ZFace accomplishes dense 3D registration from 2D video without person-specific training. Tracked faces are normalized in terms of rotation and scale and then centered. Faces then are normalized to the inter-ocular distance (IOD) of 80 pixels. We obtain $224 \times 224$ pixel images of faces with 80 pixels IOD.

**Fig. 2:**
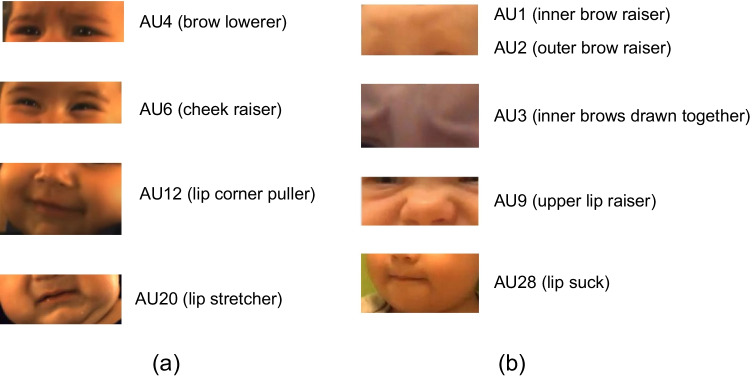
Action Units (AUs) that are automatically detected with Infant AFAR. AUs shown in Fig. 2a are manually annotated in both infant databases and the ones shown in Fig. 2b are manually annotated in only one of the databases (CLOCK)

### Action unit detection

In all cases, we use a deep learning approach that is based on training and testing a convolutional neural network. We use the VGG-16 network, which is a convolutional neural network containing 16 layers (Simonyan & Zisserman, 2014) pretrained on the ImageNet database (Deng et al., 2009), which includes 1.2 million images, as the initial network. Initializing the model with the weights of this pre-trained model has been shown to outperform initializing one with random weights for AU detection as well as other visual classification tasks (Niinuma, Jeni, Onal Ertugrul, & Cohn, 2019). Since the first few layers capture low-level information that is learned on ImageNet, the first two convolutional blocks are kept frozen before fine-tuning the remaining layers. We train individual networks for the four AUs that are common to both databases; namely, AU4, AU6, AU12, and AU20 as shown in Fig. 2a for thorough cross-domain generalizability investigations. We also train individual networks for the five additional AUs that are manually annotated only for CLOCK; namely AU1, AU2, AU3, AU9, and AU28 as shown in Fig. 2b to perform comparisons with the available AU detectors. The final layer of the VGG-16 network is replaced with a layer having a single neuron for occurrence detection of individual AUs. A sigmoid activation function is used at the output of final layer for non-linearity. We use binary-cross entropy loss (*L*) as follows:

1 $$\begin{aligned} L = y \cdot \log \hat{y} + (1 - y) \cdot \log (1 - \hat{y}) \end{aligned}$$

where y is actual AU occurrence, $\hat{y}$ is predicted occurrence.

Training is performed using stochastic gradient descent optimizer with a learning rate $lr = 10 ^{-3}$ and momentum $= 0.9$. Values obtained at the output neuron are between [0, 1], corresponding to the occurrence probability of the related AU. During test time, we assign the positive AU occurrence label to the instances with probability greater than or equal to 0.5. To avoid over fitting, we perform 3-fold cross validation in all of the experiments. Since the base rates of AUs are low in both infant databases (see Table 1), we obtain a balanced training set for each fold. We down-sample frames in which AU is absent so that to the number of frames where AU is present and absent are equal.

**Table 1:** Base rates of AUs in the infant databases

Base rates	AU1	AU2	AU3	AU4	AU6	AU9	AU12	AU20	AU28
MIAMI	−	−	−	0.10	0.31	−	0.26	0.12	−
CLOCK	0.26	0.20	0.23	0.11	0.33	0.07	0.22	0.18	0.08

### Studies

We perform five studies to evaluate the performance of infant AU detectors as follows:

**Study 1:** We train and test the AU detectors using frames from the same database (within MIAMI and within CLOCK). AU detectors are trained from scratch (i.e. network weights are randomly initialized before training). Since these databases are collected in relatively controlled environments, domains including context, illumination, and video resolution are the same or similar for different participants in the same database. Previous works have shown that AU detection performance is generally better for within database studies compared to cross database ones (Onal Ertugrul et al., 2019a; Ertugrul et al., 2020). Differences in domains may hurt the performance. Within database performance may be considered as the expected upper limit for an AU detector.

**Study 2:** We train and test the AU detectors using frames from both MIAMI and CLOCK databases. AU detectors are trained from scratch (i.e. network weights are randomly initialized before training). MIAMI and CLOCK databases differ in context (Face-to-Face/Still-Face mother-infant interaction vs. positive/negative emotion tasks with an experimenter), illumination, video resolution, and age. Infants are 4 and 13 months in MIAMI and CLOCK, respectively.

**Study 3:** We train AU detectors using frames from one infant database (e.g. MIAMI or CLOCK) and test them with the other database. Our goal is to investigate how well AU detectors trained on one infant database generalize to an unseen domain (the other infant database).

**Study 4:** We train AFAR on adult faces in EB+ (Onal Ertugrul et al., 2019a) database where age of participants range from 18 years to 66 years and GFT (Girard, Chu, Jeni, & Cohn, 2017) database where age of participants range from 21 years to 28 years. We used this adult AFAR model to detect AUs in infant faces in MIAMI and CLOCK to understand how well an AU detector trained on adult faces generalize to detect AUs in infant faces.

**Study 5: Infant AFAR:** We first train our AU detector on adult faces in databases EB+ (Onal Ertugrul et al., 2019a) (200 adult subjects) and GFT (150 adult subjects) (Girard et al., 2017) to obtain adult AFAR. Then we fine-tune adult AFAR using the frames from the infant databases to detect AUs in infant faces. The final model is referred to as Infant AFAR. The initial network captures the AU-related information from adult faces and the fine-tuning step helps learning infant-specific features related to AUs.

**Table 2:** AU detection performances on MIAMI dataset

−	S	AUC	PA	NA
(a) AU4				
Study 1: MIAMI $\rightarrow$ MIAMI	0.88	0.83	0.69	**0.97**
Study 2: (MIAMI + CLOCK) $\rightarrow$ MIAMI	0.87	0.80	0.65	0.96
Study 3: CLOCK $\rightarrow$ MIAMI	0.80	0.79	0.57	0.94
OpenFace	−0.08	0.64	0.25	0.58
Study 4 - Adult AFAR: (EB+ + GFT) $\rightarrow$ MIAMI	0.74	0.82	0.54	0.92
Study 5 - Infant AFAR: (EB+ + GFT + MIAMI + CLOCK) $\rightarrow$ MIAMI	**0.90**	**0.85**	**0.73**	**0.97**
(b) AU6				
Study 1: MIAMI $\rightarrow$ MIAMI	0.75	**0.86**	**0.81**	0.90
Study 2: (MIAMI + CLOCK) $\rightarrow$ MIAMI	0.73	**0.86**	0.80	0.89
Study 3: CLOCK $\rightarrow$ MIAMI	0.34	0.75	0.65	0.68
OpenFace	0.51	0.63	0.43	0.85
Study 4 - Adult AFAR: (EB+ + GFT) $\rightarrow$ MIAMI	0.59	0.82	0.73	0.83
Study 5 - Infant AFAR: (EB+ + GFT + MIAMI + CLOCK) $\rightarrow$ MIAMI	**0.76**	**0.86**	**0.81**	**0.91**
(c) AU12				
Study 1: MIAMI $\rightarrow$ MIAMI	0.77	0.85	0.77	**0.92**
Study 2: (MIAMI + CLOCK) $\rightarrow$ MIAMI	**0.78**	**0.87**	**0.79**	**0.92**
Study 3: CLOCK $\rightarrow$ MIAMI	0.72	0.81	0.72	0.91
OpenFace	0.67	0.74	0.63	0.89
Study 4 - Adult AFAR: (EB+ + GFT) $\rightarrow$ MIAMI	0.60	0.77	0.66	0.85
Study 5 - Infant AFAR: (EB+ + GFT + MIAMI + CLOCK) $\rightarrow$ MIAMI	0.76	0.84	0.77	0.91
(d) AU20				
Study 1: MIAMI $\rightarrow$ MIAMI	0.81	0.79	0.64	0.94
Study 2: (MIAMI + CLOCK) $\rightarrow$ MIAMI	0.81	0.81	0.66	0.94
Study 3: CLOCK $\rightarrow$ MIAMI	0.66	0.79	0.52	0.89
OpenFace	0.56	0.55	0.21	0.87
Study 4 - Adult AFAR: (EB+ + GFT) $\rightarrow$ MIAMI	−	−	−	−
Study 5 - Infant AFAR: (EB+ + GFT + MIAMI + CLOCK) $\rightarrow$ MIAMI	−	−	−	−

Left-side of the $\rightarrow$ denotes the database(s) used to train the model in the related study. Right-side of the $\rightarrow$ denotes the database used to test the models (i.e. MIAMI)

The best results are shown in bold

**Table 3:** AU detection performances on CLOCK dataset

−	S	AUC	PA	NA
(a) AU4				
Study 1: CLOCK $\rightarrow$ CLOCK	0.82	**0.79**	**0.61**	**0.96**
Study 2: (MIAMI + CLOCK) $\rightarrow$ CLOCK	0.78	0.80	0.57	0.94
Study 3: MIAMI $\rightarrow$ CLOCK	0.81	0.60	0.31	0.95
Hammal et al. (2017)	**0.84**	−	0.19	**0.96**
OpenFace	−0.01	0.62	0.26	0.62
Study 4 - Adult AFAR: (EB+ + GFT) $\rightarrow$ CLOCK	0.70	0.72	0.46	0.91
Study 5 - Infant AFAR: (EB+ + GFT + MIAMI + CLOCK) $\rightarrow$ CLOCK	0.77	0.79	0.56	0.93
(b) AU6				
Study 1: CLOCK $\rightarrow$ CLOCK	0.73	0.87	0.81	0.89
Study 2: (MIAMI + CLOCK) $\rightarrow$ CLOCK	0.75	**0.88**	**0.83**	0.90
Study 3: MIAMI $\rightarrow$ CLOCK	0.68	0.78	0.72	0.89
Hammal et al. (2017)	0.74	−	0.76	0.91
OpenFace	0.65	0.83	0.69	0.89
Study 4 - Adult AFAR: (EB+ + GFT) $\rightarrow$ CLOCK	0.67	0.77	0.69	0.89
Study 5 - Infant AFAR: (EB+ + GFT + MIAMI + CLOCK) $\rightarrow$ CLOCK	**0.77**	**0.88**	**0.83**	**0.91**
(c) AU12				
Study 1: CLOCK $\rightarrow$ CLOCK	0.78	0.86	0.76	**0.93**
Study 2: (MIAMI + CLOCK) $\rightarrow$ CLOCK	**0.80**	0.86	0.77	**0.93**
Study 3: MIAMI $\rightarrow$ CLOCK	0.74	0.80	0.70	0.92
Hammal et al. (2017)	0.77	−	0.64	**0.93**
OpenFace	0.67	0.83	0.69	0.89
Study 4 - Adult AFAR: (EB+ + GFT) $\rightarrow$ CLOCK	0.73	0.76	0.65	0.92
Study 5 - Infant AFAR: (EB+ + GFT + MIAMI + CLOCK) $\rightarrow$ CLOCK	0.79	**0.87**	**0.78**	**0.93**
(d) AU20				
Study 1: CLOCK $\rightarrow$ CLOCK	**0.72**	0.81	0.66	0.91
Study 2: (MIAMI + CLOCK) $\rightarrow$ CLOCK	**0.72**	0.83	**0.67**	0.91
Study 3: MIAMI $\rightarrow$ CLOCK	0.68	0.57	0.24	0.91
Hammal et al. (2017)	**0.72**	−	0.48	**0.92**
OpenFace	0.58	0.58	0.31	0.87
Study 4 - Adult AFAR: (EB+ + GFT) $\rightarrow$ CLOCK	−	−	−	−
Study 5 - Infant AFAR: (EB+ + GFT + MIAMI + CLOCK) $\rightarrow$ CLOCK	−	−	−	−

Left-side of the $\rightarrow$ denotes the database(s) used to train the model in the related study. Right-side of the $\rightarrow$ denotes the database used to test the models (i.e. CLOCK)

The best results are shown in bold

### Evaluation

Different metrics capture different properties of AU detection performance. We report a variety of metrics: S score (free-margin kappa), area under ROC curve (AUC), positive agreement (PA), and negative agreement (NA) following (Girard et al., 2017).

PA is computed as $\frac{2tp}{2tp+fp+fn}$. When only two methods are compared (e.g., Infant AFAR versus manual AU coding), PA is equivalent to F1 (harmonic mean of precision and recall), which is the most commonly used metric in AU detection literature. PA quantifies performance on correct predictions on positive samples. NA is the complement of PA and is computed as $\frac{2tn}{2tn + fp + fn}$. It evaluates the solution by the harmonic agreement of instances not including AUs.

Area Under the Receiver Operating Characteristics Curve (AUC) is equal to the probability that a classifier will rank a randomly chosen frame in which AU is present higher than a randomly chosen one in which AU is absent. Therefore, this measure shows the success of classifier to rank frames with and without AU.

S score or free-marginal kappa coefficient provides a chance-adjusted summary statistic (Girard et al., 2017). It is computed as $\frac{2tp + 2tn}{tp + fp + fn + tn} -1$ . It is equal to the ratio of observed non-chance agreement to possible non-chance agreement. It estimates chance agreement by assuming that each category is equally likely to be chosen at random.

Many of the AUs have low base rates. AUC is robust to imbalanced data while PA and NA are not (Jeni, Cohn, & De La Torre, 2013), which should be taken into account when evaluating results for AUs that occur less often.

## Results

We report AU detection results for four AUs that are central to emotion expression and social signaling that are common to both infant databases: AU4 (brow lowerer), AU6 (cheek raiser), AU12 (lip corner puller), and AU20 (lip stretcher). Table 2 shows results on MIAMI database. Table 3 shows results on CLOCK. Since EB+ and GFT lack annotations for AU20, Adult AFAR and Infant AFAR results are not possible for AU20.

### Comparison of the performance of infant and adult AU detectors on infant databases

Our first question is whether AU detectors trained in adult faces (i.e. Adult AFAR) generalize well to infant faces. To answer this question, we compare the performances of the infant AU detector in Study 1 (in which the same database is used to train and test the model) and adult AU detector (i.e. Adult AFAR) in Study 4. Results of the adult AU detector are low and consistently much lower compared to the results of the infant AU detector for both databases. On MIAMI dataset, PA scores for the Infant AU detectors (Study 1) are 15% higher for AU4, 8% higher for AU6, and 11% higher for AU12 compared to PA in Study 4. On CLOCK dataset, PA for the Infant AU detectors are 15% higher for AU4, 12% higher for AU6, and 9% higher for AU12 compared to PA in Study 4. Similar differences in the performances are observed in S scores and AUC values for both databases. As noted above, Infant and Adult AU detectors could not be compared for AU 20. These results suggest that models trained to detect AUs in adult faces fail to generalize well to infant faces.

### Comparison of within-database and cross-database performance

In adult databases, cross database performance is lower than within-database performance (Onal Ertugrul et al., 2019a; Ertugrul et al., 2020). Our second question is whether same is found for generalizability between infant databases. To answer this question, we compare the performances in Study 1 and Study 3.

Cross-database results are diminished compared to within-database results for all AUs and all measures. On MIAMI dataset, PA in Study 1 (within-database) are 12% higher for AU4, 16% higher for AU6, 5% higher for AU12, and 12% higher for AU20 compared to PA in Study 3 (between-database). On CLOCK dataset, PA in Study 1 are 30% higher for AU4, 9% higher for AU6, 6% higher for AU12, and 42% higher for AU20 compared to PA in Study 3. Similarly, very high differences are observed when AUC is used. These results suggest that a model trained on one infant database fails to generalize well to the other infant database. This finding is analogous to what has been found previously for adult AU detection. These findings suggest that efforts are needed to adapt models to new domains.

We further elaborate on the cross-domain performances of the models trained on MIAMI and CLOCK. For AU4 and AU20, cross-database results are very different for MIAMI and CLOCK although the within-database performances are similar. For example, when PA is used, cross-database performance to detect AU4 is 0.57 for the model trained on CLOCK and tested on MIAMI (see Table 2a) whereas 0.31 for the model trained on MIAMI and tested on CLOCK (see Table 3a). Similarly, for AU20 achieved PA for cross domain experiments is 0.52 on MIAMI (see Table 2d) and 0.24 on CLOCK (see Table 3d). It can be inferred that models trained on CLOCK generalizes better to detect AU4 and AU20 in the unseen databases (e.g. MIAMI), compared to the models trained on MIAMI. For AU12, cross-domain performances on CLOCK and MIAMI are similar. For AU6, the model trained on MIAMI and tested on CLOCK performed better (0.72 PA in Table 3b) compared to the model trained on CLOCK and tested on MIAMI (0.65 PA in Table 2b). We can infer that AU6 detectors trained on MIAMI generalize better to unseen domains.

### Comparison of Infant AFAR with infant AU detectors that are trained from scratch

Our third question is whether fine-tuning the pre-trained Adult AFAR with infant faces outperforms training infant AU detectors from scratch. For this comparison, we trained two models from scratch: (1) In Study 1, we train and test the model with the same infant database and (2) In Study 2, we train and test the model with a combination of MIAMI and CLOCK database. We compare the results obtained in Study 1 and Study 2 with the results of Infant AFAR obtained in Study 5.

Infant AFAR, in which adult AFAR is fine-tuned using infant faces, performs the best in most cases and achieves comparable performance to within database or within age-group performances in the rest. In both MIAMI and CLOCK databases Infant AFAR achieves the best performance to detect AU6 by performing better than or equal to within database results in Study 1 (which can be considered as the upper limit). Infant AFAR achieves the best performance to detect AU4 on MIAMI when all measures are used. On CLOCK, Infant AFAR performance is similar to results of Study 2 and slightly worse than results of Study 1. For AU12, Infant AFAR achieves the second best result after results of Study 2 on MIAMI but these results are very similar. On CLOCK, Infant AFAR achieves the best performance when AUC, PA and NA are used for evaluation.

Overall, fine-tuning adult AFAR with infant faces performs the best or similar to the best to detect AUs in the infant faces. We provide Infant AFAR models for AU4, AU6, and AU12 with this paper. For AU20, we provide the model trained with both MIAMI and CLOCK in Study 2.

**Table 4:** AU detection performances on CLOCK dataset (additional AUs)

−	S	AUC	PA	NA
(a) AU1				
Study 1: CLOCK $\rightarrow$ CLOCK	0.50	**0.67**	**0.51**	0.83
Hammal et al. (2017)	**0.77**	−	0.48	**0.94**
OpenFace	0.40	0.63	0.45	0.79
Study 4 - Adult AFAR: (EB+ + GFT) $\rightarrow$ CLOCK	0.15	0.61	0.46	0.65
(b) AU2				
Study 1: CLOCK $\rightarrow$ CLOCK	0.52	**0.67**	**0.46**	0.84
Hammal et al. (2017)	**0.77**	−	0.33	**0.94**
OpenFace	0.30	0.60	0.38	0.75
Study 4 - Adult AFAR: (EB+ + GFT) $\rightarrow$ CLOCK	0.44	0.62	0.40	0.82
(c) AU3				
Study 1: CLOCK $\rightarrow$ CLOCK	0.67	**0.72**	**0.58**	0.90
Hammal et al. (2017)	**0.69**	−	0.50	**0.91**
OpenFace	−	−	−	−
Study 4 - Adult AFAR: (EB+ + GFT) $\rightarrow$ CLOCK	−	−	−	−
(d) AU9				
−	S	AUC	PA	NA
Study 1: CLOCK $\rightarrow$ CLOCK	**0.86**	**0.82**	**0.55**	0.96
Hammal et al. (2017)	0.77	−	0.26	**0.98**
OpenFace	0.76	0.75	0.39	0.93
Study 4 - Adult AFAR: (EB+ + GFT) $\rightarrow$ CLOCK	−	−	−	−
(e) AU28				
Study 1: CLOCK $\rightarrow$ CLOCK	**0.84**	**0.81**	**0.57**	**0.96**
Hammal et al. (2017)	0.83	−	0.25	0.72
OpenFace	0.81	0.50	0.04	0.95
Study 4 - Adult AFAR: (EB+ + GFT) $\rightarrow$ CLOCK	−	−	−	−

The best results are shown in bold

### Comparison with the previous AU detectors

We compared the performance of Infant AFAR with an infant AU detector proposed by Hammal et al. (2017) and an open source toolbox OpenFace (Baltrusaitis et al., 2018) trained on adult faces.

Hammal et al. (2017) reported AU detection results on CLOCK database. Infant AFAR significantly outperforms the method of Hammal et al. (2017) to detect AU4 (37% improvement), AU6 (7% improvement), and AU12 (14% improvement) when PA values are compared. For AU20, our model trained on both infant databases achieved 19% improvement in PA over Hammal et al. (2017). Note that S and NA values are similar for both models except for AU4, where Infant AFAR performed 7% worse when S values are compared. These results suggest that our models are more successful to detect the AUs in the positive samples compared to Hammal et al. (2017).

We obtained results with OpenFace on both MIAMI and CLOCK databases. When PA values are compared, Infant AFAR substantially outperforms OpenFace to detect AU4 (48% improvement on MIAMI and 30% improvement on CLOCK), AU6 (38% improvement on MIAMI and 14% improvement on CLOCK) and AU12 (14% improvement on MIAMI and 9% improvement on CLOCK). For AU20, our model trained with both databases outperformed OpenFace on MIAMI (45% improvement) and CLOCK (36% improvement) databases. Similarly, Infant AFAR outperforms OpenFace on both databases when S, AUC, and NA measures are used. Note that OpenFace yields negative S scores for AU4 on both MIAMI and CLOCK databases meaning that agreement between the two raters (manual annotations and labels assigned by OpenFace) are slightly worse than chance.

### Comparison of AU detectors on additional AUs manually annotated for CLOCK

In addition to the four AUs that are annotated for both MIAMI and CLOCK, five additional AUs namely, AU1, AU2, AU3, AU9 and AU28 are manually annotated for only CLOCK database. Although thorough cross-domain experiments cannot be performed for these AUs, we can compare the performances of Study 1 (within-database), Study 4 (Adult AFAR), OpenFace, and models in Hammal et al. (2017) with the available AUs. We also make the models trained in Study 1 for the additional AUs publicly available.

Table 4 shows that when PA and AUC values are compared models trained in Study 1 yielded the best performance. When S scores are compared, the AU detector proposed by Hammal et al. (2017) outperformed our model for AU1 and AU2, both models performed similarly for AU3, and our models trained in Study 1 outperformed the AU detector by Hammal et al. (2017) for AU9 and AU28. Similarly, our models trained in Study 1 outperformed Adult AFAR and OpenFace. Note that OpenFace and Adult AFAR did not provide AU3 results. Our tool will be the first publicly available tool that provides predictions for AU3.

## Discussion and future work

AU detectors that have been trained and tested in adults are becoming available for research use (Girard, Cohn, Jeni, Lucey, & De la Torre, 2015; Onal Ertugrul et al., 2019b; Baltrusaitis et al., 2018). It may be tempting to apply them to infant faces. Our findings strongly contraindicate use of AU detectors that have not been trained and tested in infants. In the current study, state-of-the-art AU detectors trained and tested in adults greatly under-performed on all metrics when applied to infant faces. AU detectors for adults cannot be assumed valid for infants in absence of evidence to the contrary.

AU detectors when trained and tested in different infant databases may have reduced generalizability as well. Infant AFAR was trained and tested in databases that differed in head pose, illumination, video resolution, emotion context and infant age. Infant AFAR generally outperformed AU detectors trained separately within databases. These findings are consistent with what has been reported previously in adults (Ertugrul et al., 2020). Greater diversity in training data and greater similarity between training and application domains optimize performance. Diversity in training and testing data are strengths of Infant AFAR. Nevertheless, the generalizability of Infant AFAR to domains much different from the ones in which it was trained and tested is an empirical question

Pre-training on a large dataset and fine-tuning on the dataset of interest has been shown to improve performance in several machine learning tasks including speech recognition (Bansal, Kamper, Livescu, Lopez, & Goldwater, 2019), biomedical image analysis (Zhou et al., 2017) and 3D point cloud understanding (Xie et al., 2020). Consistent with AU detection results in adults, pre-training and fine-tuning optimized classifiers (Niinuma et al., 2019). Previous work in AU detection in infants has omitted pre-training in adults and fine-tuning. Infant AFAR outperformed previous state-of-the-art in infant AU detection (Hammal et al., 2018). Lack of pre-training and fine-tuning in that previous work may have been a contributing factor.

Infant AFAR is proposed to contribute to advancing behavior research on infants. Infant AFAR can automatically detect the occurrence of AUs that are central to expression of positive and negative affect. AU12 is associated with social smile and in combination with AU6 is associated with the Duchenne enjoyment smile. The combination of AU4 and AU20 is associated with cry-face and combination of AU4, AU6, and AU20 is observed during a Duchenne cry-face (Mattson et al., 2013; Kohut et al., 2012). Additionally, with the models trained only on CLOCK, Infant AFAR can detect the occurrence of AU1 (inner brow raiser), AU2 (outer brow raiser), AU3 (inner brows drawn together), AU9 (nose wrinkler), and AU28 (lip suck). One limitation of Infant AFAR is that it can detect only a limited number of AUs compared to the off-the-shelf toolboxes. This limitation is caused by the limited number of AUs manually coded using BabyFACS. Yet, it can detect a set of AUs that are observed frequently during spontaneous behavior with superior performance. These action units and smile / cry-face expressions are important to automatically investigate infant behavior in several works, including but not limited to investigating infant’s response to mother’s unresponsiveness during face-to-face / still-face protocol (Ahn et al., 2020a; Ahn et al., 2021), assessing facial nerve injuries and disorders (Hammal et al., 2018), automatically analyzing social communication behaviors in children with suspected Autism Spectrum Disorder (Ahn et al., 2020b, reaction to tastes (Rosenstein & Oster, 1988), and experience of pain (Kohut et al., 2012; Mattson et al., 2013).

## Open practices statement

The primary data are identifiable video of parents and infants (MIAMI database) or infants (CLOCK database). All parents gave informed consent to use of the video by the investigators but not to other researchers. For this reason, we regrettably are unable to make the video available to others.

Code for Infant AFAR is available through Github^1^.

## Footnotes

1 https://github.com/AffectAnalysisGroup/AFARtoolbox/tree/master/InfantAFAR
